# MicroRNAs in Chronic Kidney Disease: Four Candidates for Clinical Application

**DOI:** 10.3390/ijms21186547

**Published:** 2020-09-07

**Authors:** Linsey J. F. Peters, Jürgen Floege, Erik A. L. Biessen, Joachim Jankowski, Emiel P. C. van der Vorst

**Affiliations:** 1 Institute for Molecular Cardiovascular Research (IMCAR), RWTH Aachen University Hospital, 52074 Aachen, Germany; lipeters@ukaachen.de (L.J.F.P.); erik.biessen@mumc.nl (E.A.L.B.); jjankowski@ukaachen.de (J.J.); 2 Department of Pathology, Cardiovascular Research Institute Maastricht (CARIM), Maastricht University Medical Centre, 6229 ER Maastricht, The Netherlands; 3 Interdisciplinary Center for Clinical Research (IZKF), RWTH Aachen University Hospital, 52074 Aachen, Germany; 4 German Centre for Cardiovascular Research (DZHK), Partner Site Munich Heart Alliance, 80336 Munich, Germany; 5 Division of Nephrology and Clinical Immunology, RWTH Aachen University Hospital, 52074 Aachen, Germany; jfloege@ukaachen.de; 6 Institute for Cardiovascular Prevention (IPEK), Ludwig-Maximilians-University Munich, 80336 Munich, Germany

**Keywords:** MicroRNAs, kidney fibrosis, chronic kidney disease, clinical application

## Abstract

There are still major challenges regarding the early diagnosis and treatment of chronic kidney disease (CKD), which is in part due to the fact that its pathophysiology is very complex and not clarified in detail. The diagnosis of CKD commonly is made after kidney damage has occurred. This highlights the need for better mechanistic insight into CKD as well as improved clinical tools for both diagnosis and treatment. In the last decade, many studies have focused on microRNAs (miRs) as novel diagnostic tools or clinical targets. MiRs are small non-coding RNA molecules that are involved in post-transcriptional gene regulation and many have been studied in CKD. A wide array of pre-clinical and clinical studies have highlighted the potential role for miRs in the pathogenesis of hypertensive nephropathy, diabetic nephropathy, glomerulonephritis, kidney tubulointerstitial fibrosis, and some of the associated cardiovascular complications. In this review, we will provide an overview of the miRs studied in CKD, especially highlighting miR-103a-3p, miR-192-5p, the miR-29 family and miR-21-5p as these have the greatest potential to result in novel therapeutic and diagnostic strategies.

## 1. Introduction

Chronic kidney disease (CKD) is a major contributor to morbidity and mortality. It is assumed to become the fifth most common cause of death worldwide in the year 2040 [1]. CKD is classified into five stages based on estimated glomerular filtration rate (eGFR) (G1-5) and albumin-to-creatinine ratio (ACR) (A1-3) as shown in Table 1 and Table 2 [2]. The underlying causes of CKD genesis and progression vary and besides genetic causes include for example diabetes and hypertension resulting in diabetic kidney disease and hypertensive kidney disease, respectively. Sustained or repeated kidney injury over time leads to maladaptive responses, such as extracellular matrix (ECM) deposition in the glomeruli and tubulointerstitium. This results in the common features of CKD: tubulointerstitial fibrosis and glomerulosclerosis. These pathological changes provoke an abnormal kidney structure, hypoxia, microvascular capillary rarefaction, and tubular atrophy, which all together leads to a decrease in renal filtration capacity and eventually to end-stage kidney disease (ESKD) [3,4].

Although CKD has a great impact on health worldwide, CKD itself is generally not the direct cause of death in industrialized countries in contrast to many other parts of the world, due to effective treatment options. However, CKD impacts global health indirectly by considerably increasing the risk of cardiovascular diseases (CVDs) [5,6]. Furthermore, many studies have already clearly shown that heart failure additionally leads to kidney failure and vice versa, as reviewed elsewhere [7,8,9]. Some predisposing factors for developing CVDs and CKD are shared, such as obesity, diabetes, and age, and some risk factors for CVDs are CKD-related, such as increased uremic toxin concentrations, proteinuria, and altered mineral metabolism [10]. The most common CKD-related CVD death is ischemic heart disease, which includes myocardial infarction, angina pectoris and sudden cardiac death [11]. Ischemic heart disease accounts for approximately 55% of all CVD deaths in CKD stage 2–4 patients and for about 75% of deaths in stage 5 (ESKD) patients [12]. Another commonly found cardiac pathology in CKD patients is uremic cardiomyopathy, a term not very well defined, which is reviewed elsewhere in detail [13].

Patients with mild to moderate CKD are often asymptomatic, leaving them untreated and consequently many of these patients progress to advanced stages of CKD [14]. Currently, albuminuria and eGFR constitute the central tools for the diagnosis of CKD and its stages. However, these tools mostly enable diagnosis of CKD after kidney damage has already occurred, they do not provide etiologic clues and only limited information on disease activity in the kidneys [15]. Hence, earlier and more personalized diagnosis and therapy of CKD may be more suitable to limit the CKD-associated mortality. For this, more detailed mechanistic insights are needed into CKD development and progression. Over the last years, multiple microRNA (miR) species have been identified to play a crucial role in this pathology. This review will highlight four miRs that have been widely studied and shown potential to be used in the clinic in the future as diagnostic or even therapeutic tools.

### 1.1. MicroRNA Synthesis and Function

**Figure 1:**
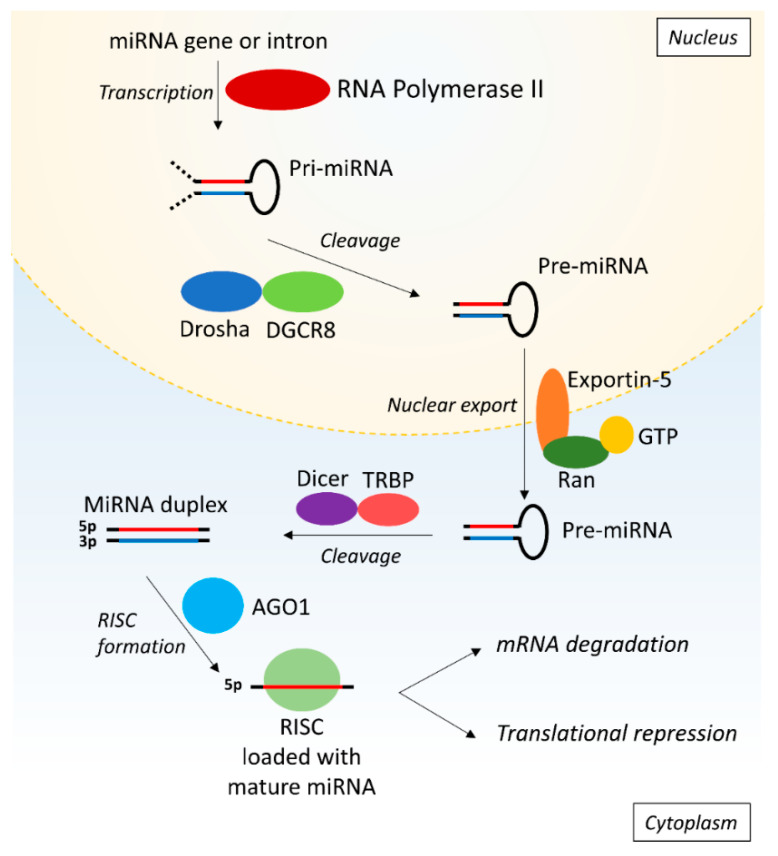
MicroRNA biosynthesis. First, the pri-miR is synthesized from the DNA by RNA polymerase II and is subsequently cleaved by Drosha and DGCR8 into pre-miR. The pre-miR is exported into the cytoplasm by Exportin-5 and Ran-GTP, followed by cleavage into a miR duplex by Dicer and TRBP. One strand is loaded into RISC consisting of AGO1, Dicer and TRBP. The RISC is guided to the target mRNA where the miR induces target degradation or translational repression of the mRNA. AGO1: argonaute 1; DGCR8: Drosha/DiGeorge syndrome critical region gene 8; pre-miR: primary microRNA; pre-mRNA: precursor messenger ribonucleic acid; Ran-GTP: GTP-binding nuclear protein Ran; RISC: RNA-induced silencing complex; RNA: ribonucleic acid; TRBP: tar RNA binding protein.

MiRs are small non-coding RNA fragments of approximately 22 nucleotides that are involved in post-transcriptional gene regulation by targeting the 3′ UTR of mRNAs [16]. The stepwise process of synthesis of miRs starts with primary miR (pri-miR) that is transcribed from the DNA by RNA polymerase II (Pol II) (Figure 1). Subsequently, the Drosha/DiGeorge syndrome critical region gene 8 (DGCR8) microprocessor complex crops the pri-miR to produce precursor miR (pre-miR). Exportin-5 and GTP-binding nuclear protein Ran (Ran-GTP) then mediate the export of the pre-miR from the nucleus into the cytoplasm where the pre-miR is processed by Dicer and Tar RNA binding protein (TRBP) eventually producing a miR duplex containing the mature 5p and 3p miR strands. In the nomenclature of miRs, the additions of ‘-5p’ or ‘-3p’ behind the specific miR number (e.g., miR-21-5p) indicate which strand is loaded into a RNA-induced silencing complex (RISC). The RISC consists of Dicer, TRBP, and Argonaute 1 (AGO1) amongst other things and is subsequently guided towards the target mRNA [17,18]. The miR binds to the 3′ UTR of mRNA if the seed sequence-a highly conserved sequence at positions 2–7 from the miR 5′ end-is a match and has either a partial or full sequential complementarity to its target, resulting in translational repression or mRNA degradation respectively [16,17]. In this manner, miRs can modulate the expression of a wide range of proteins and are implicated in various processes like cell proliferation, apoptosis, and differentiation.

### 1.2. miRs as Diagnostic Tools

Different sources of miRs can be used for biomarker research. For example, miRs can be extracted from plasma, serum, urine or exosomes, i.e., small membrane vesicles containing varies proteins, lipids, and nucleic acids [19]. MiRs are relatively stable in both serum and urine under a variety of storage conditions [20,21]. However, the stability of exosomal miRs isolated from plasma depends on the method of exosome isolation and storage, indicating the importance of optimized extraction methods [22]. If handled correctly, exosomal miRs seem to be more stable than non-exosomal miRs and therefore are preferred as in biomarker research [23]. Besides their diagnostic use, miRs could also serve as prognostic biomarkers to support physicians decision-making in therapy as reviewed in [24]. Conventional detection methods include polymerase chain reaction (PCR), microarrays, RNA sequencing (RNA-seq), and in situ hybridization (ISH), which will be briefly highlighted in this section [25].

PCR is the classical miR detection method and basically amplifies small segments of DNA. Besides regular PCR more specialized PCR methods are available like droplet digital PCR, enabling more sensitive and reproducible analyses [26,27]. However, these more specialized PCR techniques are labor intensive and costly, and thereby mostly used for non-clinical, small scale research. A major limitation of PCR is that it is hypothesis-driven as one can only check for a specific or small set of miRs.

Microarrays, i.e., biochips enabling high-throughput screening, overcome the limitations of PCR as this method can map miR expression profiles in a unbiased manner [28]. The method is expensive, but can simultaneously provide high throughput analyses of various microRNAs between various organs or tissues or between health and disease [28]. Because of their time-, cost-, and labor-intensive features, microarrays are unlikely to be routinely used in the clinic.

RNA-Seq is a method to isolate and sequence miRs in a high-throughput manner and is for example used to determine miR expression patterns. Unlike microarrays, which contain a specific set of known targets, RNA-Seq is not dependent on pre-chosen targets and has therefore an even wider coverage than microarrays. Although the method has disadvantages such as high costs and run length [29], the current, existing, and publicly available RNA-seq data sets could be used to overcome these hurdles. The existing datasets are often based on kidney biopsies from CKD patients, but have largely been analyzed to only evaluate protein coding genes. Therefore, it would be worthwhile to take another look at these datasets especially focusing on miRs, which could yield valuable information and at the same time reduce the cost of RNA-seq, assuming that the samples are prepared without poly(A) polymerase which is unfortunately often not the case.

ISH is a powerful method to directly detect and localize miR expression levels within tissue or individual cells. Of all miR detection techniques, ISH is the only method providing spatial information. To increase specificity and stability, locked nucleic acids probes are used for this method [30], which are RNA derivative nucleotides in which the ribose ring is locked in a specific conformation.

### 1.3. miRs as Therapeutic Tools

Therapeutically, miR levels have to be either raised or their binding capacity to mRNAs has to be decreased, depending on the desired effects [31,32]. Oligonucleotide manipulation techniques, including antagomiRs and locked nucleic acids, are already widely employed to inhibit miR binding to mRNA in research settings or clinical trials [31,33]. Besides miR targeting, small interfering RNAs (siRNAs) are also promising therapeutic agents [34]. SiRNAs and miRs share many similarities as both induce gene silencing at the post-transcriptional level, though their mechanisms of action are distinct. The major difference between these two short duplex RNA molecules is that siRNAs target one mRNA, whereas miRs can have multiple targets [34].

AntagomiRs are modified oligonucleotides and have characteristic complementary sequences, which extend beyond the seed sequence. They can thereby scavenge the miR away from its target mRNA [35]. Furthermore, miR expression can be inhibited by locked nucleic acids, which have an exceptional binding affinity that allows to use short sequences complementary to just the seed region of the miR [36]. Finally, miR mimics can be used to elevate miR levels [37]. These double-stranded RNA fragments act similarly to the targeted miR and bind to the same mRNA target, thereby increasing the potential to modulate the translation process [37].

Increasing miR levels with mimics or inhibiting miRs by antagomiRs is already being evaluated in clinical trials [38,39] in, for example, indications such as cancer or hepatitis C [40,41,42]. All of these trials showed some improvements in the clinical outcome of treated patients, but are still ongoing to assess their long-term efficacy and the safety of miR interference in particular [39].

## 2. miRs in Kidney Disease

A large number of miRs has been identified that may play a role in CKD, although the exact relevance in the majority of cases currently remains elusive. Table 3 summarizes current insights into the involvement of miRs in CKD, with a special emphasis on hypertensive nephropathy, Table 4 on diabetic kidney disease/diabetic nephropathy and Table 5 on kidney tubulointerstitial fibrosis. Additionally, Table 6 highlights the effects that these miRs have in CKD-associated CVD. The role of miRs in other kidney diseases—such as acute kidney injury, glomerulonephritides, or systemic autoimmune diseases—have been reviewed elsewhere [43,44,45,46,47], and will therefore not be discussed in the current review.

Several studies have focused on changes in miR expression patterns during the various stages of CKD, including ESKD, kidney transplantation, and hemodialysis patients [48,49,50]. Since this topic has been covered in other reviews [51,52], it will not be further discussed here.

This review will highlight the four miRs, which, based on data of therapeutically relevant animal models and clinical studies, have been identified as the most promising ones to be used in the clinic in the future as diagnostic or even therapeutic tools in the context of CKD and associated CVD.

### 2.1. miR-103a-3p

Recently, Lu et al. investigated the role of miR-103a-3p in angiotensin-II-induced kidney inflammation and fibrosis [55]. Angiotensin-II exerts vasoconstrictive effects and pro-inflammatory actions on post-glomerular arteries, resulting in glomerular injury and kidney fibrosis [133,134]. Although the pathological role of angiotensin-II in hypertensive nephropathy is well established [135], the underlying molecular mechanisms remain poorly understood. Over the last years, some miRs have been associated with hypertensive nephropathy (Table 3), although most of these studies only demonstrated changes in miR expression upon disease development and progression and did not elucidate whether miRs themselves have a causal role in disease development. Comparing patients with hypertensive nephropathy to normotensive healthy controls, Lu et al. demonstrated that both serum and urine miR-103a-3p levels were significantly higher in hypertensive nephropathy patients [55]. Additionally, when mice were injected with different doses of angiotensin-II, this increased urine and serum miR-103a-3p levels independently of effects on arterial blood pressure.

To further study the role of miR-103a-3p in angiotensin-II-induced kidney injury, mice that either overexpressed miR-103a-3p by using a recombinant adeno-associated virus or mice with a knockdown of miR-103a-3p by the means of a locked nucleic acid-anti-miR-103a-3p were investigated [55]. Overexpression of miR-103a-3p in angiotensin-II-infused mice resulted in albuminuria, kidney inflammation, and kidney fibrosis, whereas knockdown of miR-103a-3p resulted in decreased angiotensin-II-induced effects on the kidney. These effects were mediated by SNRK, which was identified as a novel target of miR-103a-3p. In line with the fact that miR-103a-3p downregulates *Snrk* expression, kidneys of patients with hypertensive nephropathy expressed lower levels of SNRK compared to control kidneys. Further supporting this mechanism, it was shown that angiotensin-II infusion in mice reduced SNRK levels in a miR-103a-3p dependent manner.

All in all, this study identified a novel miR-103a-3p/SNRK dependent mechanism by which angiotensin-II induces kidney injury. Although the role of miR-103a-3p in the development of hypertensive nephropathy has been clearly demonstrated, it would be interesting to perform further studies to elucidate whether miR-103a-3p knockdown in already established hypertensive nephropathy can prevent further progression of the disease. Such studies would be required to validate miR-103a-3p as new therapeutic target in hypertensive and potentially other nephropathies.

### 2.2. mir-192-5p

One essential miR, involved in the development of diabetic kidney disease (Table 4), is miR-192-5p. Overexpression of miR-192-5p in proximal tubular epithelial cells suppressed the expression of ZEB1 (also known as δEF1) and ZEB2 (also known as SIP1) in-vitro, thereby opposing the TGF-β-mediated downregulation of E-cadherin [89]. Further supporting the role of miR-192-5p in fibrosis is the finding that increased abundance of miR-192-5p reduced the expression of ZEB1/2 and thereby increased collagen 1 alpha 2 (*Col1a2*) expression in mesangial cells [85].

The in-vivo relevance of the above findings was confirmed in mouse models of type 1 (streptozotocin (STZ)-injection) and type 2 (*db*/*db*) diabetes. Compared to non-diabetic control mice, the expression of miR-192-5p was significantly increased in isolated glomeruli from diabetic mice, and this coincided with increased levels of TGF-β1 and Col1a2 [85]. TGF-β1-induced miR-192-5p expression is also relevant in the context of glomerular mesangial cell survival and hypertrophy [87]. These effects are mediated by a complex mechanism in which reduced ZEB2 resulted in increased expression of miR-216a-5p/miR-217-5p, thereby inhibiting phosphatase and tensin homolog (PTEN) and increasing Akt expression and causing mesangial cell hypertrophy [87,136]. Interestingly, it could also be observed that a positive feedback loop exists in this system as miR-192-5p upregulated TGF-β1 [91]. This feedback loop in turn was at least partly driven by miR-200b/c-3p, which was identified as a downstream target of miR-192-5p.

The in-vivo relevance of miR-192-5p in diabetic nephropathy was assessed further in mice with a genetic deletion of miR-192-5p [90]. STZ-injected miR-192-5p deficient mice displayed attenuated kidney TGF-β1 expression and had reduced kidney fibrosis, glomerular hypertrophy, and albuminuria compared to diabetic wild-type mice [90]. To evaluate the therapeutic potential of targeting miR-192-5p, locked nucleic acid-anti-miR-192-5p was tested in a mouse model of STZ-induced diabetic nephropathy [86]. In the kidneys of diabetic mice, inhibition of miR-192-5p significantly increased ZEB1/2 expression, while decreasing the expression of TGF-β1, collagen, and fibronectin, resulting in less kidney fibrosis and attenuated albuminuria. This further supports the notion that locked nucleic acid-anti-miR-192-5p is an interesting approach for the prevention or perhaps even treatment of diabetic kidney disease [86].

Interestingly, in contrast to the animal studies described above, in a cohort of patients with established diabetic kidney disease, miR-192-5p expression was lowest in kidney biopsies of patients with advanced diabetic kidney disease [89]. In addition, low levels of kidney miR-192-5p correlated with tubulointerstitial fibrosis and impaired eGFR [89]. Analyses of extracellular vesicles isolated from urine of patients with type 2 diabetes demonstrated that the expression of miR-192-5p was significantly increased in microalbuminuric patients compared to normoalbuminuric diabetics and healthy controls. Notably, miR-192-5p expression levels dropped significantly again in macroalbuminuric patients. These findings suggest that miR-192-5p could also play a disease stage-dependent role in diabetic kidney disease, which needs further confirmation [88]. This could explain the apparently controversial findings between the in vivo animal studies and the human study, as the animal models present a less advanced disease stage than the patients. Furthermore, receiver operating characteristic (ROC) curve analyses revealed that miR-192-5p had an area under the curve (AUC) of 0.802 in discriminating the normoalbuminuric from microalbuminuric patients [88].

Besides its role in diabetic kidney disease, miR-192-5p has also been implicated in non-diabetic kidney fibrosis (Table 5). Kidney miR-192-5p was enriched in mice with unilateral ureteral obstruction (UUO) and rats with 5/6 nephrectomy, both models of progressive kidney fibrosis [137]. In vitro, in rat tubular epithelial cells, miR-192-5p stimulated TGF-β1-induced collagen expression [119]. Additionally, TGF-β1 also induced miR-192-5p expression, which was shown to be mediated via Smad3. Finally, in-vitro studies revealed that miR-192-5p targeting may have therapeutic potential, as treatment of rat tubular epithelial cells with a miR-192-5p inhibitor blocked TGF-β1-induced collagen production [119]. Taken together, these data suggest that miR-192-5p might play a causal role in non-diabetic kidney fibrosis and CKD as well.

### 2.3. miR-29 Family

The miR-29 family consists of three members, miR-29a-3p, miR-29b-3p and miR-29c-3p. While all three family members share a common seed sequence, they also have unique sequence features causing functional differences [138]. In a mouse model of early STZ-induced diabetic nephropathy, the kidney expression of all three miR-29 family members was decreased compared to non-diabetic controls [79]. In advanced diabetic nephropathy, induced by uninephrectomy plus STZ-injection in rats, both kidney miR-29a-3p and miR-29c-3p expression was significantly decreased [79]. MiR-29 family members repress the expression of TGF-β1 and the collagen genes *col1a2* and *col4a1* in proximal tubular cells, podocytes, as well as mesangial cells [79]. As for miR-192-5p, the actions of miR-29 family members are mediated via Smad3, and knockdown of miR-29b-3p in mesangial cells enhanced TGF-β1/Smad3-mediated fibrosis in-vitro [77]. The decreased kidney miR-29b-3p expression diabetic nephropathy mice (*db*/*db*) also coincided with increased microalbuminuria, kidney fibrosis, and kidney inflammation [77]. In line with this and strongly supporting a role of miR-29b-3p in DN, overexpression of miR-29b-3p by ultrasound-microbubble-mediated gene transfer normalized microalbuminuria and attenuated kidney injury [77]. In contrast to the animal studies, a human study in two independent cohorts of diabetic patients demonstrated that the urinary expression of miR-29b-3p was increased in diabetic kidney disease patients compared to control subjects [76]. This further highlights the importance of the location at which expression is measured, as in all the animal studies miR-29 expression was measured in kidney tissue while in the human cohort its expression was measured in urine samples.

MiR-29 family members also play a role in the development of non-diabetic CKD. In different CKD models (adenine nephropathy, UUO) miR-29 expression decreased upon disease development [79,109,110]. As in experimental diabetic nephropathy, overexpression of miR-29b-3p using ultrasound-microbubble-mediated gene transfer again prevented intrakidney collagen III upregulation and kidney fibrosis in a mouse model of UUO [109]. These beneficial effects were confirmed by intramuscular injection of exosomes containing miR-29a/b/c-3p into mice with UUO, resulting in an attenuation of kidney fibrosis and decreased blood urea levels, which again seemed to be mediated by reduced TGF-β3 and collagen production [111].

### 2.4. miR-21-5p

The role of miR-21-5p in diabetic kidney disease was supported by studies that showed increased expression of miR-21-5p in serum or kidney of diabetic kidney disease patients or rodents with diabetic nephropathy [62,64,65,66,68,69]. This increased expression in diabetic kidney disease and diabetic nephropathy was correlated with tubulointerstitial fibrosis, kidney injury, and lower eGFR [62,64,65,68,69].

In-vivo targeting of miR-21-5p using locked nucleic acid, ultrasound-microbubble-mediated gene transfer or lentiviral vectors has been used to validate its role in the development of DN. Knockdown of miR-21-5p by using locked nucleic acid-anti-miR-21-5p in STZ-induced murine diabetes, resulted in decreased interstitial fibrosis due to reduced expression of the collagen genes *Col1a2* and *Col III* [69]. MiR-21-5p knockdown also ameliorated kidney inflammation, namely infiltration by F4/80 positive macrophages, possibly via reducing the expression of monocyte chemotactic protein-1 (MCP-1), a well-known chemokine that mainly attracts monocytes and macrophages to sites of injury [69]. Additionally, miR-21-5p silencing prevented podocyte loss and reduced albuminuria compared to control mice [69]. Comparable results were obtained using an approach, where miR-21-5p was knocked down using ultrasound-microbubble-mediated gene transfer [65]. Transfer of miR-21-5p knockdown plasmids into kidneys of diabetic *db*/*db* mice ameliorated kidney fibrosis as well as overexpression of TGF-β1, collagen I and IV. Furthermore, expression of the pro-inflammatory cytokines tumor necrosis factor-α (TNF-α) and MCP-1 was reduced upon miR-21-5p knockdown, which coincided with a reduced infiltration of F4/80 positive macrophages [65].

Using various in-vitro studies, Smad7 was identified as a key underlying regulator of both miR-21-5p induced fibrosis and inflammation [65]. Overexpression of miR-21-5p in rat tubular epithelial cells and mesenchymal cells decreased Smad7 expression, while knockdown of miR-21-5p restored Smad7 levels [65]. Furthermore, knockdown of Smad7 in-vitro increased the expression of inflammatory cytokines, collagen, and fibronectin, confirming that Smad7 plays a protective role in kidney fibrosis and inflammation [65]. Several other studies confirmed these effects of miR-21-5p on kidney fibrosis and inflammation in diabetic nephropathy models, for example, inhibition of miR-21-5p in a mouse model (KK-Ay mice) by lentiviral vectors [67] and a mouse (KK-Ay mice) and rat (STZ injection) model in which suppression of miR-21-5p via antagomiR-21-5p reduced kidney fibrosis and inflammation [66,68]. These studies targeting miR-21-5p clearly establish the potential to halt experimental diabetic nephropathy but it is unknown so far whether targeting of miR-21-5p also has therapeutic potential for prevention or reversal of DN.

The impact of miR-21-5p on non-diabetic kidney fibrosis has been tested in the UUO and the unilateral ischemia reperfusion injury (IRI) model using mice genetically lacking miR-21-5p or treated with anti-miR-21-5p oligonucleotides [107]. The lack or silencing of miR-21-5p in both models ameliorated albuminuria, kidney fibrosis and injury [107]. Similarly, ultrasound-microbubble-mediated gene transfer of miR-21-5p-knockdown plasmids in mice with UUO reduced the development of kidney fibrosis [106]. Finally, the therapeutic potential of this targeting approach was assessed by knocking down miR-21-5p in established kidney fibrosis by injecting the miR-21-5p knockdown plasmid 4 days after UUO. Under these circumstances, miR-21-5p-knockdown also halted the progression of fibrosis [106], suggesting that miR-21-5p targeting may become a new therapeutic option to combat kidney fibrosis.

Alport syndrome is a genetic disorder caused by inherited mutations in *COL4A3*, *COL4A4* and *COL4A5* genes. Its key features include hearing loss, glomerulonephritis and ESKD [139]. Mice deficient in *Col4a3* have been used as a murine model for Alport nephropathy [140]. The expression level of miR-21-5p was elevated in the kidney, glomeruli, and tubules of these mice compared to controls. Additionally, miR-21-5p expression was increased from 3 weeks of age in *Col4a3^−/−^* kidneys and increased further along with disease progression. In the same study, *Col4a3^−/−^* mice were injected subcutaneously with anti-miR-21 oligonucleotides [140]. Inhibition of miR-21-5p resulted in significantly lower albuminuria and blood urea nitrogen (BUN), which reflect improved kidney function, as compared to vehicle control. Notably, anti-miR-21 treatment prolonged median survival of mice by 32 days and did not show any adverse side-effects. Moreover, miR-21-5p silencing enhanced PPARα/retinoid X receptor (PPARα/RXR) activity, which was likely the cause of the observed protection against TGF-β-induced fibrogenesis and inflammation in glomerular and interstitial cells [140]. Tubular function was also preserved by miR-21-5p silencing by enhanced mitochondrial function resulting in reduced ROS production [140]. Another pre-clinical study showed the association of miR-21-5p with Alport syndrome in dogs. Dogs suffering from X-linked hereditary nephropathy (XLHN) were used in this study as it is the equivalent to human Alport syndrome. Kidney samples were harvested at several time points during disease progression, including ESKD. In XLHN dogs, kidney miR-21-5p was first significantly upregulated at the time point at which serum creatinine was increased [141]. Additionally, kidney miR-21-5p expression levels were correlated with clinical markers of kidney function. More specifically, miR-21-5p expression was positively correlated with the serum creatinine concentration, whilst it was negatively correlated with GFR [141]. The expression pattern of miR-21-5p has also been examined in the kidneys of Alport syndrome patients [142]. The miR expression was significantly increased in kidney samples from Alport syndrome patients compared to controls and miR-21-5p levels positively correlated with serum BUN, serum creatinine, proteinuria, and overall severity of kidney pathology. MiR-21-5p was also shown to be localized to damaged tubular epithelial cells and glomeruli. Furthermore, human kidney samples of patients with Alport syndrome showed abnormal expression of genes involved in fibrosis, mitochondrial function, inflammation, and kidney injury [142]. Worth mentioning are the clinical trials focusing on miR-21-5p in Alport syndrome. A phase I and phase II clinical trial included the usage of the drugs RG012 and SAR339375, respectively to target miR-21-5p. Despite their potential and interesting set-up, the trials were delayed due to re-organization within companies and have yet to publish results (NCT03373786 and NCT02855268). Overall, both animal and human studies suggest that miR-21-5p plays a pivotal role in the progression of CKD caused by Alport syndrome. It also suggests that therapeutic interference of this miR may become a new way of battling the consequences of Alport syndrome.

Besides these kidney effects, miR-21-5p also impacts CKD-related cardiovascular effects (Table 6). For example, in 5/6 nephrectomized rats concentric left-ventricular hypertrophy, not driven by volume expansion, ensued within 4 to 5 weeks after CKD induction [130]. Additionally, the lipid content in left ventricular tissue increased at week 7 after surgery. Although there was no overall left ventricular fibrosis, perivascular fibrosis increased at 7 weeks post CKD induction. At this time point, small RNA deep sequencing in left ventricular tissue revealed an upregulation of miR-21-5p. To study the significance of increased left ventricular miR-21-5p expression, anti-miR-21-5p oligonucleotides were injected intravenously into 5/6-nephrectomized and sham operated rats at week 1 and 4 post-surgery. Suppression of miR-21-5p reduced left ventricular wall thickening and improved cardiac function, without significant effects on blood pressure or the kidney pathology, indicating that the changes in cardiac phenotype were not driven by a reduction in kidney injury [130]. This lack of kidney effects of miR-21-5p suppression is in sharp contrast with the kidney studies discussed above and is likely caused by substantial differences in disease models and treatment protocols.

Further analysis revealed altered peroxisome proliferator-activated receptor alpha (PPARα) expression in the left ventricle upon miR-21-5p suppression. Involvement of PPARα was further demonstrated by delivering a low dose of the PPARα agonist clofibrate via daily injections. Clofibrate treatment in nephrectomized rats prevented left ventricular hypertrophy and improved cardiac function, similar to rats treated with anti-miR-21-5p. This study therefore unveiled a new pathway involved in mediating CKD-related cardiovascular effects.

## 3. Limitations and Future Perspectives

In this review, we discussed the role of miRNA in CKD and highlighted four miRs that have the potential to serve as novel biomarkers and therapeutic targets in CKD and associated CVDs. Besides these specific miRs, many more miRs have been demonstrated to play a role in CKD. Although miRs are currently not yet being used in such a manner in the clinic, this review highlights their enormous potential and thereby the need for further pre-clinical and especially clinical research in this field. However, before miRs can be routinely used as biomarkers or therapeutics in the clinic, first some crucial aspects need to be addressed in future research.

One important aspect which should be taken into consideration is the fact that the number of miRs varies between species. The human genome contains more than 2500 miRs, while rats have less than 1000 and mice a little less than 2000 miRs [143]. In order to enable proper translation into the clinic, one should already in a pre-clinical stage select the miRs, which are highly conserved between species.

Furthermore, it is necessary to determine the optimal miR detection method and in which type of sample the miRs should be measured. As shown before, different methods are being used—e.g., RT-PCR, RNA-seq, or ISH—which all have specific advantages and disadvantages. Additionally, studies have either used serum, plasma, or urine samples or even only isolated exosomes from these liquids. In order to optimize miR use as a biomarker, pre-clinical studies should focus on all of these potential sources to select the most optimal one to be validated in the clinic. On another note, in kidney diseases urine seems to have a great advantage over blood as a source for biomarkers. Urine has direct access to the damaged tissues of the kidney and urinary tract, making it a closer, non-invasive source of biomarkers than peripheral blood.

Moreover, a common note is the wide range of targets of miRs. A single miR is known to be able to regulate several mRNA targets and a single mRNA target can be regulated by several miRs. These targets are regulated and expressed in a tissue- and even cell-dependent manner. Thus, if one would target these miRs with mimics or antagomiRs, the chances of inducing off-target effects would be greatly increased as compared to standard drugs. To reduce these side-effects researchers should focus on methods to deliver miR therapeutics to specific tissues or even cells. Currently, carefully engineered nanoparticles are being studied for this purpose. These particles are coated with antigens that can be recognized by receptors specifically expressed on the targeted cells. Although promising, nanoparticle treatment needs to be optimized as some obstacles, such as low efficiencies in cell internalization and in the release of antagomiRs into the cytoplasm, remain [144].

Another note of caution for researchers is the variability between patients. Variables such as age, gender, and ethnicity can already have an impact on the biomarker profile and response to therapy. Other variables such as the use of medication or having comorbidities could also affect biomarker results or treatment efficiency. Furthermore, related to therapies, disease conditions have been shown to increase pharmacological effects of antagomiRs [145]. The disease severity and type could therefore influence therapeutic actions of miR-related therapies. However, these variables might actually make the use of miRs as biomarkers and therapeutics highly attractive, as it adds to a common goal in medicine: personalized medicine.

**Figure 2:**
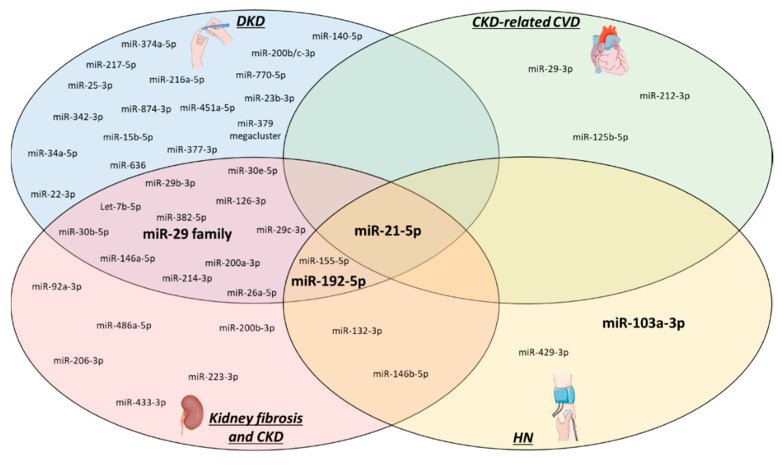
CKD-related microRNAs. All miRs that have until now been studied in CKD (ex-vivo or in-vivo models only) are depicted based on their relation to the specific pathologies. From all of these, the four relevant miRs that are highlighted in this review are visualized in bold. HN: hypertensive nephropathy, DKD: diabetic kidney disease, CVD: cardiovascular disease, CKD: chronic kidney disease.

Besides the limitations mentioned above, our review unveiled another area in CKD-related miR research that needs to be addressed. Figure 2 provides an overview of the miRs already studied in kidney fibrosis, CKD (including diabetic and hypertensive) and CKD-related CVD. As clearly visible in the figure, miR research has primarily focussed on DKD, kidney fibrosis and CKD, while CKD-related CVD and HKD have received comparably little attention up until now. Future studies should therefore focus on these neglected areas, especially since CVD and CKD are interlinked with one another and represent a high burden on global health. Another interesting aspect to highlight in Figure 2 is the overlap between the subtypes of CKD and CKD-related CVD, like for example miR-21-5p which is involved in all four described pathologies. It is worthwhile to research miRs that could connect two or more CKD subtypes, as it might unveil important common targets, underlying pathways or biomarkers.

## 4. Conclusions

Various therapies are being employed to slow down the progression of CKD [146]. However, none of the current therapies completely halt progression and none are capable of reversing the disease [147]. Although, miRs are currently not yet being used in the clinic, this review highlights four miRs, which have the potential for clinical application in the near future like the antagonism of miR-21-5p in diabetic nephropathy or the locked nucleic acid-anti-miR-103a-3p in hypertensive nephropathy. However, these studies investigate the effect of miR modulation on the initial development of CKD, rather than the real therapeutic effects of miR modulation on already established diseases, but clearly demonstrate the potential of miR modulation using agomiRs and antagomiRs. Currently, better methods for synthesis and delivery of miR are being developed to provide safer and more accurate delivery, such as lipid-based nanoparticles, FDA-approved poly-lactic-co-glycolic acid (PLGA)-based nanoparticles and ultrasound microbubble-mediated gene transfer [148,149]. Such methods are required to enable human clinical trials based on miR targeting strategies to fully evaluate its therapeutic potential.

All in all, it has been suggested that miRs can play an important role in a clinical diagnostic setting, although validation studies are still required [150]. Therefore, MiRs might have a significant beneficial impact on present-day diagnostic and therapeutic strategies and they bear great potential in CKD and associated CVD in the near future.

## Figures and Tables

**Table 1:** Estimated glomerular filtration rate (eGFR) categories in CKD.

Category	eGFR (mL/min/1.73 m^2^)	Terms
**G1**	≥90	Normal or high
**G2**	60–89	Mildly decreased
**G3a**	45–59	Mildly to moderately decreased
**G3b**	30–44	Moderately to severely decreased
**G4**	15–29	Severely decreased
**G5**	<15	Kidney failure

Abbreviations: eGFR: estimated glomerular filtration rate.

**Table 2:** Albuminuria categories in CKD.

Category	AER (mg/24 h)	ACR (mg/mmol)	ACR (mg/g)	Terms
**A1**	<30	<3	<30	Normal to mildly increased
**A2**	30–300	3–30	30–300	Moderately increased
**A3**	>300	>30	>300	Severely increased

Abbreviations: AER: albumin excretion rate; ACR: albumin-to-creatinine ratio.

**Table 3:** Overview of miRs that are involved in hypertensive nephropathy (only ex-vivo/in-vivo evidence).

miR	Targeted Genes/Pathway	Observed Effects	MiR-Effect on Disease	Change in miR-Level	Model	Species	Ref
miR-21-5p	n.d.	Kidney expression was increased in DOCA-salt treated mice (male; aged ±12–13 weeks)	n.a.	Increase	Ex-Vivo	Mouse	[53]
		miR/creatinine ratio in urine 4 days after DOCA-salt treatment was higher compared to controls and preceded albuminuria (male; aged ±12–13 weeks)	n.a.	Increase	Ex-Vivo	Mouse	
miR-21-5p	PPARα	Inhibition using a lentivirus in Ang II-treated mice ameliorated albuminuria and kidney fibrosis without lowering BP (male; aged 14 weeks)	Detrimental	n.a.	In-Vivo	Mouse	[54]
miR-103a-3p	SNRK	Upregulated in urine/serum of AngII-infused mice, independent of blood pressure, and correlated positively with albuminuria (aged ±12 weeks)	n.a.	Increase	Ex-Vivo	Mouse	[55]
		Overexpression using AAV system increased albuminuria, kidney inflammation fibrosis (aged ±12 weeks)	Detrimental	n.a.	In-Vivo	Mouse	
		Inhibition using LNA-anti-miR reduced Ang-II induced kidney inflammation and injury (aged ±12 weeks)	Detrimental	n.a.	In-Vivo	Mouse	
		Serum and urine levels were increased in a cohort of HN patients and correlated positively with albuminuria (aged 18–60 years)	n.a.	Increase	Ex-Vivo	Human	
miR-132-3p	n.d.	Kidney expression increased in DOCA-salt treated mice (male; aged ±12–13 weeks)	n.a.	Increase	Ex-Vivo	Mouse	[53]
miR-146b-5p	n.d.	Kidney expression increased in DOCA-salt treated mice (male; aged ±12–13 weeks)	n.a.	Increase	Ex-Vivo	Mouse	[53]
miR-155-5p	n.d.	Kidney expression increased in DOCA-salt treated mice (male; aged ±12–13 weeks)	n.a.	Increase	Ex-Vivo	Mouse	[53]
miR-192-5p	n.d.	Kidney expression decreased in hypertensive nephrosclerosis	n.a.	Decrease	Ex-Vivo	Human	[56]
miR-429-3p	n.d.	Ang-II reduced kidney expression of miR-429-3p (male; aged ±9 weeks)	n.a.	Decrease	Ex-Vivo	Rat	[57]
		Lentiviral overexpression blocked Ang-II induced epithelial-to-mesenchymal transition and fibrogenesis without affecting BP (male; aged ±9 weeks)	Beneficial	n.a.	In-Vivo	Rat	

Abbreviations: AAV: adeno-associated virus; Ang-II: angiotensin-II; BP: blood pressure; DOCA: deoxycorticosterone acetate; HN: hypertensive nephropathy; LNA: locked nucleic acid; MiR: microRNA; n.a.: not applicable; n.d.: not determined; PPAR: peroxisome proliferator activated receptor; SNRK: sucrose non-fermentable-related serine/threonine-protein kinase.

**Table 4:** Overview of miRs that are involved in diabetic kidney disease/diabetic nephropathy (only ex-vivo/in-vivo evidence).

miR	Targeted Genes/Pathway	Observed Effects	MiR-Effect on Disease	Change in miR-Level	Model	Species	Ref
Let-7b-5p	Col1a2/4a1	Expression decreased in the kidney cortex or glomeruli of STZ-induced diabetic mice and *db*/*db* mice (aged ±18–28 weeks and ±17–19 weeks)	n.a.	Decrease	Ex-Vivo	Mouse	[58,59]
miR-15b-5p	BCL-2	Urinary expression increased in *db*/*db* mice	n.a.	Increase	Ex-Vivo	Mouse	[60]
		Increased urine levels in type 2 diabetic patients correlated with more rapid decline in kidney function	n.a.	Increase	Ex-Vivo	Human	
miR-15b-5p	n.d.	Upregulated in urine pellets and urine exosomes in patients with DKD	n.a.	Increase	Ex Vivo	Human	[61]
miR-21-5p	PTEN	Elevated levels in kidney cortex of OVE26 type 1 diabetic mice associated with increased fibronectin content (aged 3 months)	n.a.	Increase	Ex-Vivo	Mouse	[62]
miR-21-5p	n.d.	Urinary exosomal expression upregulated in DKD patients. Expression levels negatively correlated with eGFR (male and female; age average 60–72 years)	n.a.	Increase	Ex-Vivo	Human	[63]
miR-21-5p	TGF-β, SMAD7 PTEN	Levels positively correlated with tubulointerstitial fibrosis and negatively correlated with eGFR in DKD patients	Detrimental	n.a.	Ex-Vivo	Human	[64]
		Kidney expression up-regulated in STZ-induced diabetic rats	n.a.	Increase	Ex-Vivo	Rat	
miR-21-5p	SMAD7	Kidney expression increased in *db*/*db* mice and associated with microalbuminuria, kidney fibrosis and inflammation (male; Aged 20 weeks)	n.a.	Increase	Ex-Vivo	Mouse	[65]
		Gene transfer of knockdown plasmids into the diabetic kidneys of *db*/*db* mice at 10 weeks of age ameliorated microalbuminuria and kidney fibrosis and inflammation at 20 weeks of age (male; aged 20 weeks)	Detrimental	n.a.	In-Vivo	Mouse	
miR-21-5p	TIMP3	Expression upregulated in serum and kidney tissues of DKD patients (male and female; age average 52–54 years)	n.a.	Increase	Ex-Vivo	Human	[66]
		Depletion inhibited inflammation and alleviated kidney damages in STZ-induced DN rats (aged 16 weeks)	Detrimental	n.a.	In-Vivo	Rat	
miR-21-5p	SMAD7	Inhibition via lentiviral vectors reduced kidney interstitial fibrosis in DN mice and improved kidney function	Detrimental	n.a.	In-Vivo	Mouse	[67]
miR-21-5p	SMAD7	Serum and kidney expression are increased in DN mice (male; aged 12–24 weeks)	n.a.	Increase	Ex-Vivo	Mouse	[68]
		Serum levels positively correlated with albuminuria plus kidney fibrosis and negatively with eGFR in diabetic mice (male; aged 12–24 weeks)	n.a.	Increase	Ex-Vivo	Mouse	
		Fibrosis reduced after antagomiR-21-5p treatment (male; aged 12–24 weeks)	Detrimental	n.a.	In-Vivo	Mouse	
miR-21-5p	Cdc25a Cdk6	Serum expression increased in diabetic patients. Correlated with tubulointerstitial injury in kidney biopsies of patients (male and female; Age average 58–64 years)	n.a.	Increase	Ex-Vivo	Human	[69]
		Kidney expression increased in STZ-induced diabetic mice (male; aged ±16–18 weeks)	n.a	Increase	Ex-Vivo	Mouse	
		Antagonism in STZ-induced diabetic mice decreased mesangial expansion, interstitial fibrosis, macrophage infiltration, podocyte loss, albuminuria, plus fibrosis and inflammatory gene expression (male; aged ±16–18 weeks)	Detrimental	n.a.	In-Vivo	Mouse	
miR-22-3p	PTEN	Expression increased in kidneys of STZ-induced DN rats (male)	n.a.	Increase	Ex-Vivo	Rat	[70]
miR-23b-3p	G3BP2	Serum of patients with diabetes or DKD showed reduced expression (male and female; Age average 33–52 years)	n.a.	Decrease	Ex-Vivo	Human	[71]
		Kidneys of animals with type 1 or 2 diabetes (STZ-induced and *db*/*db*) have low expression levels (male; aged 9–10 weeks)	n.a.	Decrease	Ex-Vivo	Mouse	
		Overexpression in *db/db* mice reversed hyperalbuminuria and kidney fibrosis. Antagomir treatment promoted kidney fibrosis and increased albuminuria in wild-type mice (male; aged 9–10 weeks)	Beneficial	n.a.	In-Vivo	Mouse	
miR-25-3p	CDC42	Serum of patients with diabetes or DKD showed reduced expression	n.a.	Decrease	Ex-Vivo	Human	[72]
		Kidneys of animals with type 1 or 2 diabetes (STZ-induced and *db*/*db*) have low expression levels (male; aged 9 weeks)	n.a.	Decrease	Ex-Vivo	Mouse	
		Agomir treatment in *db*/*db* mice repressed glomerular fibrosis and reduced BP. Knockdown in mice by antagomir increased proteinuria, extracellular matrix accumulation, and hypertension with renin-angiotensin system activation (male; aged 9 weeks)	Beneficial	n.a.	In-Vivo	Mouse	
miR-25-3p	NOX4	Kidney expression reduced in kidneys from STZ-induced diabetic rats (male; ±16 weeks)	n.a.	Decrease	Ex-Vivo	Rats	[73]
miR-25-3p	NOX4	Mature levels decreased in glomeruli of STZ-induced diabetic mice (aged ±12 weeks)	n.a.	Decrease	Ex-Vivo	Mouse	[74]
miR-26a-5p	CTGF	Glomerular expression downregulated in STZ-induced diabetic mice (male; aged 12 weeks)	n.a.	Decrease	Ex-Vivo	Mouse	[75]
		Expression in microdissected glomeruli positively correlated with eGFR in patients with DKD	Beneficial	n.a.	Ex-Vivo	Human	
miR-29b-3p	n.d.	Urinary expression increased in patients with DKD (male and female; age average 52–62 years)	n.a.	Increase	Ex-Vivo	Human	[76]
miR-29b-3p	TGF-β SMAD3	Kidney expression decreased in db/db mice; associated with progressive microalbuminuria, kidney fibrosis, inflammation (male; aged 10–20 weeks)	n.a.	Decrease	Ex-Vivo	Mouse	[77]
		Overexpression by ultrasound-based gene therapy attenuated DN (male; aged 10–20 weeks)	Beneficial	n.a.	In-Vivo	Mouse	
miR-29c-3p	Spry1	Levels significantly increased in the kidney glomeruli of (*db*/*db* and STZ-induced) diabetic mice (aged 24 weeks)	n.a.	Increase	Ex-Vivo	Mouse	[78]
		Knockdown by antisense oligonucleotide reduced albuminuria and kidney mesangial matrix content in *db*/*db* mice (aged 8–22 weeks)	Detrimental	n.a.	In-Vivo	Mouse	
miR-29 family	Col1a2 Col4a1	Kidney expression decreased in STZ-induced diabetic mice with early diabetic kidney fibrosis (aged ±18 weeks)	n.a.	Decrease	Ex-Vivo	Mouse	[79]
		MiR29a-3p and miR-29c-3p kidney expression decreased in unilateral nephrectomized STZ-diabetic rats characterized by advanced diabetic RF and associated with increased fibrosis (male; aged ±28 weeks)	n.a.	Decrease	Ex-Vivo	Rat	
miR-30b-5p	n.d.	Urinary exosomal expression downregulated in DKD patients. Levels positively correlated with eGFR (male and female; age average 60–72 years)	n.a.	Decrease	Ex-Vivo	Human	[63]
miR-30e-5p	n.d.	Expression downregulated in plasma and urine of patients with DKD (male and female; age average 21–30 years)	n.a.	Decrease	Ex-Vivo	Human	[80]
miR-34a-5p	GAS1	Expression increased in diabetic *db*/*db* mice (male; aged 12 weeks)	n.a.	Increase	Ex-Vivo	Mouse	[81]
		Down-regulation using antagomir alleviated glomerular hypertrophy (male; aged 12 weeks)	Detrimental	n.a.	In-Vivo	Mouse	
miR-34a-5p	n.d.	Upregulated in urine pellets and urine exosomes in DKD patients	n.a.	Increase	Ex Vivo	Human	[61]
miR-126-3p	n.d.	Urinary expression was increased in patients with DKD (male and female; age average 52–62 years)	n.a.	Increase	Ex-Vivo	Human	[76]
miR-140-5p	TLR4	Downregulated in kidney and peripheral blood from patients with DKD. Levels inversely correlated with proteinuria	n.a.	Decrease	Ex-Vivo	Human	[82]
miR-146a-5p	n.d.	In STZ-induced diabetes, miR-146a^−/−^ mice showed significantly exacerbated proteinuria, kidney macrophage infiltration, glomerular hypertrophy, fibrosis, increased M1 macrophage phenotype, and inflammasome activation (aged ±19–28 weeks)	Beneficial	n.a.	In-Vivo	Mouse	[83]
miR-146a-5p	n.d.	Kidney expression increased in patients with DKD (male and female; age average 46–51)	n.a.	Increase	Ex-Vivo	Human	[84]
		During the induction and progression of the disease in type 1 and type 2 DN rat models, expression increased gradually (male; aged ±7–14 weeks)	n.a.	Increase	Ex-Vivo	Rat	
miR-155-5p	n.d.	Kidney expression increased in patients with DKD and correlated negatively with eGFR (male and female; age average 46–51)	n.a.	Increase	Ex-Vivo	Human	[84]
		During the induction and progression of the disease in type 1 and type 2 DN rat models, expression increased gradually (male; aged ±7–14 weeks)	n.a.	Increase	Ex-Vivo	Rat	
miR-155-5p	n.d.	Urinary expression was increased in patients with DKD (male and female; age average 52–62 years)	n.a.	Increase	Ex-Vivo	Human	[76]
miR-192-5p	SIP1 (ZEB2)	Levels enhanced in glomeruli isolated from STZ-injected diabetic mice as well as diabetic *db*/*db* mice relative to corresponding nondiabetic controls, in parallel with increased TGF-β and Col1a2 levels (aged ±17 weeks)	n.a.	Increase	Ex-vivo	Mouse	[85]
miR-192-5p	Zeb1/2	In kidneys of STZ-induced diabetic mice, inhibition by LNA-anti-MiR increased Zeb1/2 and decreased expression of collagen, TGF-*β*, fibronectin and thereby kidney fibrosis. LNA-antimiR attenuated proteinuria in diabetic mice (male; aged ±12–27 weeks)	Detrimental	n.a.	In-Vivo	Mouse	[86]
miR-192-5p	Zeb2	LNA-antimiR treatment of mice resulted in decreased miR-216a-5p, miR-217-5p and Col1a2 expression in kidneys (aged ±17 weeks)	Detrimental	n.a.	In-Vivo	Mouse	[87]
miR-192-5p	n.d.	ROC curve analysis revealed that urinary EV expression had an AUC of 0.802 in discriminating the normoalbuminuric group from patients with DKD. Urinary EV levels positively correlated with albuminuria levels and TGF-*β*1 expression (male and female; age average 55–59 years)	Detrimental	n.a.	Ex-Vivo	Human	[88]
miR-192-5p	Zeb1/2	Lower expression in kidney biopsies patients with advanced DKD. Low expression correlated with increased tubulointerstitial fibrosis and low eGFR (male and female; age average 49–56 years)	Beneficial	Decrease	Ex-Vivo	Human	[89]
miR-192-5p	Zeb2	Deficiency attenuated kidney cortical TGF-β expression, kidney fibrosis, hypertrophy, proteinuria, and albuminuria in STZ-induced diabetic mice (aged 22 weeks)	Detrimental	n.a.	In-Vivo	Mouse	[90]
miR-192-5p	TGF-β, Zeb2	Inhibition decreased the expression of miR-200b/c-3p, *Col1a2*, *Col4a1*, and *TGF*-*β1* in mouse kidney cortex (aged ±17–20 weeks)	Detrimental	n.a.	Ex-Vivo	Mouse	[91]
miR-200a-3p	n.d.	Expression decreased in kidneys from mice with early and advanced diabetic kidney disease (aged ±12–18 weeks)	n.a.	Decrease	Ex-Vivo	Mouse	[92]
miR-200b/c-3p	Zeb1	Expression increased in glomeruli from type 1 (STZ-induced) and type 2 (*db*/*db*) diabetic mice (aged ±17–20 weeks)	n.a.	Increase	Ex-Vivo	Mouse	[91]
miR-200b/c-3p	FOG2	Levels significantly increased in glomeruli of *db*/*db* mice as well as STZ mice (aged 26–28 weeks)	n.a.	Increase	Ex-Vivo	Mouse	[93]
miR-214-3p	PTEN	Expression increased in isolated glomeruli from db/db mice (male; aged 18 weeks)	n.a.	Increase	Ex-Vivo	Mouse	[94]
		Treatment with a lentivirus-packed inhibitor ameliorated albuminuria and glomerular hypertrophy in *db*/*db* mice (male; aged 18 weeks)	Detrimental	n.a.	In-Vivo	Mouse	
miR-216a-5p	PTEN	Levels increased in kidney glomeruli from STZ-injected and db/db diabetic mice (aged ±17 weeks)	n.a.	Increase	Ex-Vivo	Mouse	[87]
miR-217-5p	PTEN	Expression increased in kidney glomeruli from STZ-injected and *db*/*db* diabetic mice (aged ±17 weeks)	n.a.	Increase	Ex-Vivo	Mouse	[87]
miR-342-3p	SOX6	Expression in the kidney tissues of mice with DN down-regulated (male; aged ±17–22 weeks)	n.a.	Decrease	Ex-Vivo	Mouse	[95]
miR-374a-5p	MCP-1	Kidney expression downregulated whereas MCP-1 was upregulated in tissue from DKD patients (male and female; aged 38–67)	n.a.	Decrease	Ex-Vivo	Human	[96]
miR-377-3p	SOD1/2, PAK1	Expression upregulated in kidneys from STZ-induced diabetic mice (aged ±18 weeks)	n.a.	Increase	Ex-Vivo	Mouse	[97]
miR-379-5p	LIN28B	Expression downregulated in kidneys from *db*/*db* mice (male; aged 12 weeks)	n.a.	Decrease	Ex-Vivo	Mouse	[98]
		Agomir attenuated urine protein levels, glomerular hypertrophy, mesangial amplification, kidney fibrosis in *db*/*db* mice (male; aged 12 weeks)	Beneficial	n.a.	In-Vivo	Mouse	
miR-379 mega-cluster	EDEM3, ATF3, TNRC6B, CPEB4, PHF21A	Megacluster, composed of 40 miRs, upregulated in glomeruli of STZ-induced and *db*/*db* diabetic mice (male; aged ±14–22 weeks)	n.a.	Increase	Ex-Vivo	Mouse	[99]
		Inhibition of the entire cluster via antagomiRs attenuated early DN features in mice (male; aged ±15–17 weeks)	Detrimental	n.a.	In Vivo	Mouse	
miR-382-5p	FoxO1	Expression significantly upregulated in kidney tissues of STZ-induced DN mice (male; aged ±20 weeks)	n.a.	Increase	Ex-Vivo	Mouse	[100]
miR-451a	LMP7	Downregulated in the kidneys of *db*/*db* mice and in PBMCs of patients with DKD	n.a.	Decrease	Ex-Vivo	Human	[101]
		Treatment with agomir attenuated urinary microalbumin excretion, inflammation and glomerular injury in *db*/*db* mice	Beneficial	n.a.	In-Vivo	Mouse	
miR-451a-5p	n.d.	Urinary exosome expression increased in STZ-induced diabetic rats. Urinary exosome levels at 6 weeks after induction correlated with albuminuria at 9 weeks (male; aged ±23 weeks)	n.a.	Increase	Ex-Vivo	Rat	[102]
		Kidney expression reduced in STZ-induced diabetic rats and negatively associated with degree of kidney injury (male; aged ±23 weeks)	n.a.	Decrease	Ex-Vivo	Rat	
miR-636	n.d.	Upregulated in urine pellets and urine exosomes in DKD patients	n.a.	Increase	Ex Vivo	Human	[61]
miR-770-5p	TIMP3	Kidney expression increased in DKD patients	n.a.	Increase	Ex-Vivo	Human	[103]
miR-874-3p	TLR4	Kidney expression downregulated in STZ-induced DN rats (male; aged 24 weeks)	n.a.	Decrease	Ex-Vivo	Rat	[104]
		Overexpression attenuated the inflammatory response and alleviated kidney injury in DN rats (male; aged 24 weeks)	Beneficial	n.a.	In-Vivo	Rat	

Abbreviations: ACR: urine albumin creatinine ratio; ATF3: activating transcription factor 3; AUC: area under the curve; BP: blood pressure; BCL-2: B cell lymphoma 2; Ccr: creatinine clearance ratio; Cdc25a: cell division cycle 25a; Cdk6: cyclin-dependent kinase 6; CPEB4: cytoplasmic polyadenylation element binding protein 4; CTGF: connective tissue growth factor; DN: diabetic nephropathy; DKD: diabetic kidney disease; ECM: extracellular matrix; EDEM3: ER degradation-enhancing alpha-mannosidase-like 3; eGFR: estimated glomerular filtration rate (mL/min per 1.73 m^2^); EV: extracellular vesicle; FOG2: friend of GATA2; FoxO1: Forkhead transcription factor O1; G3BP2: Ras GTPase-activating protein SH3 domain-binding protein 2; GAS1: growth arrest-specific 1; IS: indoxyl sulfate; LMP7: large multifunctional protease 7; LNA: locked nucleic acid; MCP-1: monocyte chemoattractant protein-1; MiR: microRNA; n.a.: not applicable; n.d.: not determined; NOX4: NADPH oxidase 4; PAK1: p21/Cdc42/Rac1-activated kinase 1; PBMC: peripheral blood mononuclear cell; PHF21A: PHD finger protein 21A; PTEN: phosphatase and tensin homologue; RF: kidney fibrosis; ROC: receiver operating characteristics; SIP1: Smad-interacting protein 1; SMAD: mothers against decapentaplegic homolog; SOD: superoxide dismutase; SOX6: SRY-box 6; Spry1: Sprouty homolog 1; STZ: streptozotocin; TGF-β: transforming growth factor-β; TIMP3: tissue inhibitors of metalloproteinase 3; TLR: Toll-like receptor.

**Table 5:** Overview of miRs that are involved in kidney fibrosis and CKD (only ex-vivo/in-vivo evidence).

miR	Targeted Genes/Pathway	Observed Effects	MiR-Effect on Disease	Change in miR-Level	Model	Species	Ref
Let-7b-5p	Col1a2/4a1	Expression decreased in the kidney cortex of mice with adenine-induced kidney fibrosis (aged ±12 weeks)	n.a.	Decrease	Ex-Vivo	Mouse	[59]
miR-21-5p	n.d.	Urinary exosomal expression upregulated in CKD patients. miR-21-5p levels negatively correlated with eGFR (male and female; age average 60–72 years)	n.a.	Increase	Ex-Vivo	Human	[63]
miR-21-5p	n.d.	Urinary exosome levels increased in CKD patients with and without diabetes and correlated negatively with eGFR	n.a.	Increase	Ex-Vivo	Human	[105]
miR-21-5p	SMAD3	Smad3-deficient mice were protected from upregulation of miR-21-5p and fibrosis in mice with UUO (aged ±11 weeks)	Detrimental	n.a.	In-Vivo	Mouse	[106]
		Ultrasound-microbubble-mediated gene transfer knockdown halted kidney fibrosis in established obstructive nephropathy (aged ±11 weeks)	Detrimental	n.a.	In-Vivo	Mouse	
miR-21-5p	PPARα	Highly elevated in fibrotic kidneys in a UUO and IRI model	n.a.	Increase	Ex-Vivo	Mouse	[107]
		*MiR-21-5p*^−/−^ mice had reduced interstitial fibrosis, which was phenocopied in wild-type mice treated with anti-miR oligonucleotides (male and female; aged ±10–14 weeks)	Detrimental	n.a.	In-Vivo	Mouse	
miR-26a-5p	CTGF TGF-β1	Expression decreased in kidney and serum exosomes of mice with UUO (male; aged ±10 weeks)	n.a.	Decrease	Ex-Vivo	Mouse	[108]
		Exosome-delivered miR-26a-5p attenuated UUO-induced kidney fibrosis (male; aged ±10 weeks)	Beneficial	n.a.	In-Vivo	Mouse	
miR-29b-3p	TGF-β pathway SMAD3	Expression downregulated in kidneys from mice with UUO (male; aged ±7–9 weeks)	n.a.	Decrease	Ex-Vivo	Mouse	[109]
		Gene transfer prevents RF in mice with UUO; mimic in mice with established UUO nephropathy prevented disease progression (male; aged ±7–9 weeks)	Beneficial	n.a.	In-Vivo	Mouse	
miR-29c-3p	Sp1	Expression remarkably reduced in tubular epithelial cells of rats with UUO (male; aged ±9 weeks)	n.a.	Decrease	Ex-Vivo	Rat	[110]
miR-29 family	Col1a2/4a1	In an adenine-induced model (advanced nondiabetic), kidney expression decreased and associated with increased fibrosis (aged ±12 weeks)	n.a.	Decrease	Ex-Vivo	Mouse	[79]
miR-29 family	TGF-β3	Intramuscular injection of miR-containing exosomes attenuated kidney fibrosis in mice with UUO and decreased blood urea levels (aged ±8–11 weeks)	Beneficial	n.a.	In-Vivo	Mouse	[111]
miR-30b-5p	n.d.	Urinary exosomal expression downregulated in DKD patients. Levels positively correlated with eGFR (male and female; age average 60–72 years)	n.a.	Decrease	Ex-Vivo	Human	[63]
miR-30e-5p	UCP2	Downregulated in epithelial tubular cells from kidneys of mice with UUO (male; aged ±8–11 weeks)	n.a.	Decrease	Ex-Vivo	Mouse	[112]
miR-92a-3p	SIRT1 KLF2 KLF4	Serum levels increased in patients with CKD and inversely correlated with eGFR. Serum expression positively correlated with serum IS in patients with ESKD undergoing hemodialysis (male and female; age average 52–54 years)	n.a.	Increase	Ex-Vivo	Human	[113]
		Increased levels in aortas, serum, and CD144^+^ endothelial microparticles of rats with CKD (male; aged ±17–18 weeks)	n.a.	Increase	Ex-Vivo	Rat	
miR-126-3p	n.d.	Expression negatively associated with disease progression and mortality rate in patients with CKD (male and female; age average 30–73 years)	n.a.	Decrease	Ex-Vivo	Human	[48]
miR-132-3p	TGF-β STAT3, ERK	Increased during pericyte-to-myofibroblast formation and kidney injury (aged 13 weeks)	n.a.	Increase	Ex-Vivo	Mouse	[114]
		Silencing decreased kidney fibrosis in mice with UUO. Antagomir reduced number of proliferating interstitial myofibroblasts (aged 13 weeks)	Detrimental	n.a.	In-Vivo	Mouse	
miR-146a-5p	n.d.	Administration of miR-containing nanoparticles enhanced kidney miR expression, while inhibiting kidney fibrosis and inflammation (male; aged ±9 weeks)	Beneficial	n.a.	In-Vivo	Mouse	[115]
miR-146a-5p	n.d.	Kidney expression increased in B6.MRLc1 CKD mice and correlated with kidney inflammatory cell content and cytokine levels (female)	n.a.	Increase	Ex-Vivo	Mouse	[116]
miR-146b-5p	TGF-β	miR-146b^−/−^ exacerbated kidney hypertrophy and fibrosis after 5/6 nephrectomy in female rats (male and female)	Detrimental	n.a.	In-Vivo	Rat	[117]
miR-155-5p	PDE3A	Expression increased in kidney tissues of mice with UUO (male; aged ±9–10 weeks)	n.a.	Increase	Ex-Vivo	Mouse	[118]
		Inhibition decreased kidney fibrosis and improved kidney function (male; aged ±9–10 weeks)	Detrimental	n.a.	In-Vivo	Mouse	
miR-192-5p	TGF-β	Kidney expression increased upon kidney fibrosis, induced by UUO (aged ±9 weeks)	n.a.	Increase	Ex-Vivo	Mouse	[119]
miR-200a-3p	Zeb1/2	Expression downregulated at the early phase of UUO (male; aged ±8–10 weeks)	n.a.	Decrease	Ex-Vivo	Rat	[120]
miR-200a-3p	n.d.	Expression decreased in kidneys from CKD mice (aged ±12–18 weeks)	n.a.	Decrease	Ex-Vivo	Mouse	[92]
miR-200b-3p	Zeb1/2	Kidney expression increased in a time-dependent manner mice with UUO (aged ±8–9 weeks)	n.a.	Increase	Ex-Vivo	Mouse	[121]
		Precursor injection inhibited the increase of collagen types I, III and fibronectin in obstructed kidneys, thus ameliorating fibrosis (aged ±8–9 weeks)	Beneficial	n.a.	In-Vivo	Mouse	
miR-206-3p	ANXA1	Overexpression (mimic) decreased glomerular interstitial fibrosis and tubular epithelial-to-mesenchymal transition (aged ±25 weeks)	Beneficial	n.a.	In-Vivo	Rat	[122]
miR-214-3p	mt-*Nd6* mt-*Nd4l*	Tubular expression increased in kidney tissue from patients with CKD, and positively correlated with proteinuria and kidney fibrosis	n.a.	Increase	Ex-Vivo	Human	[123]
		Kidney expression increased in mouse models of CKD induced by obstruction, ischemia/reperfusion, and albumin overload	n.a.	Increase	Ex-Vivo	Mouse	
		Proximal tubule–specific deletion attenuated apoptosis, inflammation, fibrosis, and mitochondrial damage in CKD mouse models	Detrimental	n.a.	In-Vivo	Mouse	
miR-214-3p	n.d.	Genetic deletion or treatment with anti-miR attenuated interstitial fibrosis induced by UUO (male; aged 9 weeks)	Detrimental	n.a.	In-Vivo	Mouse	[124]
		Expression detected in cells of the glomerulus and tubules in human kidneys (male; aged 9 weeks)	n.a.	n.a	Ex-Vivo	Mouse	
miR-223-3p	n.d.	Expression negatively associated with disease progression and mortality rate in patients with CKD (male and female; age average 30–73 years)	n.a.	Decrease	EX-Vivo	Human	[48]
miR-382-5p	KLK5 HSPD1	Kidney expression increased in mice with UUO (male; aged ±9 weeks)	n.a.	Increase	Ex-Vivo	Mouse	[125,126]
		LNA-anti-miR treatment attenuated interstitial fibrosis in mice with UUO (male; aged ±9 weeks)	Detrimental	n.a.	In-Vivo	Mouse	
miR-433-3p	Azin1	Kidney expression upregulated in mice with UUO (aged ±9–10 weeks)	n.a.	Increase	Ex-Vivo	Mouse	[127]
		Kidney knockdown by ultrasound microbubble–mediated gene transfer suppressed fibrosis in a prevention and intervention study (aged ±9–10 weeks)	Beneficial	n.a.	In-Vivo	Mouse	
miR-486a-5p	FoxO1	Expression decreased in tibialis anterior muscles of CKD mice (male; aged ±14 weeks)	n.a.	Decrease	Ex-Vivo	Mouse	[128]
		Transfection of muscles from CKD mice with mimic increased muscle mass (male; aged ±14–16 weeks)	Beneficial	n.a.	In-Vivo	Mouse	

Abbreviations: ANXA1: Annexin A1; Azin1: antizyme inhibitor 1; AUC: area under the curve; CKD: chronic kidney disease; CTGF: connective tissue growth factor; ECM: extracellular matrix; eGFR: estimated glomerular filtration rate (mL/min per 1.73 m^2^); ERK: extracellular-signal regulated kinase; ESKD: end-stage kidney disease; FoxO1: Forkhead transcription factor O1; HSPD1: heat shock protein 60; IRI: ischemia reperfusion injury; IS: indoxyl sulfate; KLF: Krüppel-like factor; KLK5: kallikrein 5; LNA: locked nucleic acid; MiR: microRNA; n.a.: not applicable; n.d.: not determined; PDE3A: phosphodiesterase 3A; PPAR: peroxisome proliferator activated receptor; RF: kidney fibrosis; ROC: receiver operating characteristics; SIRT1: sirtuin-1; SMAD: mothers against decapentaplegic homolog; Sp1: specificity protein 1; STAT3: signal transducer and activator of transcription 3; TGF-β: transforming growth factor-β; UCP2: uncoupling protein 2; UUO: unilateral ureteral obstruction.

**Table 6:** Overview of miRs that are involved in CKD-related CVD (only ex-vivo/in-vivo evidence).

miR	Targeted Genes/Pathway	Observed Effects	MiR-Effect on Disease	Change in miR-Level	Model	Species	Ref
miR-21-5p	n.d.	Expression increased in hearts after MI (male; aged ±24 weeks)	n.a.	Increase	Ex-Vivo	Rat	[129]
		Treatment with AST-120, a uremic toxin adsorbent, attenuated increased miR levels and cardiac fibrosis (male; aged ±24 weeks)	Detrimental	n.a.	In-Vivo	Rat	
miR-21-5p	PPARα	Upregulation in left ventricles of CKD rats (male; aged 12–17 weeks)	n.a.	Increase	Ex-Vivo	Rat	[130]
		AntagomiR improved left ventricle function and prevented left ventricle hypertrophy, but did not influence kidney pathology (male; aged 12–17 weeks)	Detrimental	n.a.	In-Vivo	Rat	
miR-29-3p	TGF-β1liuwenCol1a1	Expression levels decreased in hearts after MI (male; aged ±24 weeks)	n.a.	Decrease	Ex-Vivo	Rat	[129]
		Treatment with AST-120, a uremic toxin adsorbent, attenuated cardiac fibrosis and decreased miR expression levels (male; aged ±24 weeks)	Beneficial	n.a.	In-Vivo	Rat	
miR-125b-5p	n.d.	Aortic and serum expression decreased in rats with CKD	n.a.	Decrease	Ex-Vivo	Human	[131]
		Serum levels inversely correlated with the severity of VC and high serum levels associated with a lower risk of VC	Beneficial	n.a.	Ex-Vivo	Human	
		ESKD patients with high baseline serum levels had lower risk of VC progression after 2 years	Beneficial	n.a.	Ex-Vivo	Human	
miR-212-3p	n.d.	Left ventricular expression elevated in CKD (5/6 nephrectomy) rats whilst FoxO3 expression remained unchanged (male; aged ±18 weeks)	n.a.	Increased	Ex-Vivo	Rat	[132]

AUC: area under the curve; CKD: chronic kidney disease; ESKD: end-stage kidney disease; FoxO3: Forkhead transcription factor O1; HFpEF: heart failure with preserved ejection fraction; MI: myocardial infarction; n.a.: not applicable; n.d.: not determined OR: odds ratio; PPAR: peroxisome proliferator activated receptor; TGF-β: transforming growth factor-β; VC: vascular calcification.
